# Evaluation of a Simple and Accurate Method for Intraocular Lens Constant Optimization Using Linear Interpolation

**DOI:** 10.3390/jcm14134543

**Published:** 2025-06-26

**Authors:** Sumitaka Miyamoto, Kazutaka Kamiya

**Affiliations:** 1 Aira Miyamoto Eye Clinic, Aira 8995213, Japan; airamiyamotoganka@yahoo.co.jp; 2 School of Rehabilitation Sciences, Showa Medical University, Yokohama 2268555, Japan

**Keywords:** intraocular lens power calculation, intraocular lens constant optimization, Linear interpolation, EVO, Hill-RBF, Kane, ARGOS, OA-2000

## Abstract

**Objectives**: We devised a simple and practical method for optimizing intraocular lens (IOL) constants using linear interpolation, based on the IOL power calculation study protocol proposed by Hoffer et al., and evaluated its effectiveness. **Methods**: This retrospective study included 188 eyes from 188 Japanese patients who underwent cataract surgery with the implantation of CNA0T0 (Alcon) between June 2022 and September 2024. Preoperative biometric data were obtained using ARGOS (Alcon) and OA-2000 (Tomey). Predicted refractions were calculated using the European Society of Cataract and Refractive Surgeons’ (ESCRS) IOL Web Calculator with the EVO, Hill-RBF 3.0 (Hill), and Kane formulas, using both A-constants of 119.1 and 119.33. The mean prediction error (MPE) was calculated as the difference between the predicted and postoperative spherical equivalent at 3 months. Linear interpolation was applied to the paired results to derive optimized A-constants yielding MPE = 0 and to correct each case’s predicted refraction values (“corrected values”). Additionally, predicted refractions were recalculated using the optimized A-constants with the ESCRS IOL Web Calculator to obtain “actual values”. Both corrected and actual values achieved an MPE of 0 and were compared using the Friedman test and Cochran’s Q test. **Results**: The optimized A-constants for ARGOS were 119.540 (EVO), 119.733 (Hill), and 119.563 (Kane); for OA-2000, they were 119.388, 119.532, and 119.417, respectively. No significant differences were found between corrected and actual values under any condition. **Conclusions**: This method is simple, accurate, and applicable to new IOLs, devices, and formulas, with potential to improve the precision of clinical IOL power calculations.

## 1. Introduction

More than half a century has passed since the first intraocular lens (IOL) power calculation formula was introduced by Fedorov et al. in 1967 [1]. In recent years, advances in ocular biometry have enabled more accurate axial length (AL) measurements, as well as improved assessments of parameters such as anterior chamber depth (ACD) and lens thickness (LT) [2]. As a result, several new IOL power calculation formulas that incorporate these biometric parameters have been developed, including the Barrett Universal II formula (BU-II) [3] and the EVO formula [4]; the Hill-RBF 3.0 formula [5], which utilizes artificial intelligence (AI); and the Kane formula [6], which incorporates gender and also utilizes AI. Numerous studies evaluating the predictive accuracy of these formulas have been conducted worldwide [7,8,9,10,11].

In earlier studies, comparisons between formulas often included data from various IOL models, different surgeons, and both eyes of the same patients, potentially introducing confounding factors. To address this, a standardized study protocol for IOL power calculation was proposed by K.J. Hoffer and colleagues, which has since gained global acceptance as a methodological benchmark [12,13].

Among the recommendations in this protocol, optimizing IOL constants to achieve a mean prediction error (MPE) of 0 is particularly emphasized. However, such optimization is rarely performed in clinical settings, and the methods are not standardized. The protocol further recommends selecting one eye per patient with good postoperative visual acuity, optimizing the constant to achieve MPE = 0, and then comparing performance using metrics such as the mean absolute error (MAE) and median absolute prediction error (MedAE). For formulas that are not publicly available—such as the EVO and Kane formulas—constant optimization cannot be performed using standard software like Microsoft Excel. In such cases, optimization typically requires contacting the formula developers or using specialized programming techniques. These requirements pose a significant barrier for physicians working in small private clinics to participate in IOL power calculation research. Although many of these modern formulas are highly accurate, the implementation of custom constant optimization is typically limited to advanced users or large institutions with access to proprietary tools or technical expertise.

Therefore, this study aimed to devise a simple method that is implementable in Microsoft Excel, using the linear interpolation (LI) method to optimize IOL constants across various calculation formulas, in accordance with the standardized protocol for IOL power calculation proposed by Hoffer et al., and to verify its accuracy and clinical utility. To our knowledge, few studies have focused specifically on the methodology of IOL constant optimization.

## 2. Materials and Methods

### 2.1. Study Design

This single-center, retrospective, observational study included patients who underwent cataract surgery with the implantation of a Clareon monofocal IOL (CNA0T0, Alcon) at our institution between June 2022 and September 2024 (253 cases; 392 eyes). This study was approved on 25 October 2024 by the Kyoto Expert Ethics Committee, an ethics committee registered with the Ministry of Health, Labour and Welfare of Japan (approval ID: KEEC-21022). All patient data were anonymized, and personal information was protected. Patients were given the opportunity to opt out of this study.

### 2.2. Setting

The biometric parameters used included AL, central corneal thickness (CCT), horizontal corneal diameter (HCD; white-to-white, WTW), ACD, LT, and keratometry (K) values. Each eye in this study was measured using both ARGOS and OA-2000 on the same day.

Predicted refractions were calculated using the European Society of Cataract and Refractive Surgeons’ (ESCRS) IOL Web Calculator (accessed between December 2024 and January 2025). The recommended A-constants in the calculator were 119.1 for EVO, 119.26 for Hill-RBF, and 119.33 for Kane. In this study, we calculated predicted refractions using the EVO, Hill-RBF, and Kane formulas with two A-constants: 119.1 and 119.33.

The postoperative observation period was set at 3 months. The testing of corrected visual acuity (CVA) and subjective refraction values, including spherical and cylindrical components, was conducted at a distance of 5 m by an orthoptist, and the results were converted to spherical equivalent refraction values. Prediction errors were then computed by subtracting the predicted refraction values from the postoperative spherical equivalent refraction for each device. To avoid any bias or inadvertent intervention in clinical decision-making, IOL power calculations using the study formulas were not performed until after the completion of the observation period for all included cases.

### 2.3. IOL Power Calculation Formula Protocol

The following three IOL power calculation formulas were used in this study, with each calculated using A-constants.

#### 2.3.1. EVO (Emmetropia Verifying Optical) Formula

Developed by Tun Kuan Yeo from Singapore in 2019, the EVO formula is a thick-lens formula based on the theory of emmetropization. It generates an “emmetropia factor” for each eye and can accommodate variations in IOL geometry and power [4,8,9,10].

#### 2.3.2. Hill-RBF (Hill Radial Basis Function) Formula

Introduced in 2016 by Warren E. Hill, MD, the Hill-RBF formula is the first purely AI-based formula. It uses radial basis functions and a data-driven approach to IOL power prediction. Developed in MATLAB, it employs pattern recognition and advanced data interpolation techniques. Hill-RBF 2.0 was based on more than 12,000 eyes, while version 3.0 (available since September 2020) expanded the dataset to over 30,000 eyes [5,8,9,10,11].

#### 2.3.3. Kane Formula

The Kane formula was developed by Jack X. Kane, MD, from Australia in 2017 using data from approximately 30,000 highly accurate cases. It combines theoretical optics, thin-lens equations, and “big data” methods. As a hybrid model, it integrates optical theory with both regression and artificial intelligence elements to enhance predictive accuracy [6,8,9,10,11].

### 2.4. Device Protocol

This study used ARGOS (Alcon Laboratories, Geneva, Switzerland) version 1.6.0, which utilizes swept-source optical coherence tomography (SS-OCT) with a wavelength of 1060 nm and a 20 nm bandwidth to record the two-dimensional optical coherence tomography (OCT) data of the full eye. Different biometry parameters, from the corneal surface to the retinal pigment epithelium, are measured with the OCT, considering different refractive indices: 1.376 for the cornea, 1.336 for the aqueous and vitreous fluids, and 1.410 for the lens. The AL for ARGOS is calculated as the sum of these parameters. Keratometry (K) is obtained from OCT images using a 2.2 mm diameter ring comprising 16 LEDs, with a corneal refractive index of 1.3375 applied for a precise corneal curvature measurement [14,15,16,17,18,19].

Meanwhile, OA-2000 (Tomey, Nagoya, Japan) version 4H1/7P, which was used in this study, is based on Fourier-domain technology and utilizes SS-OCT with a variable-wavelength laser at 1060 nm to perform 41 horizontal A- and V-scans. All parameters, including ACD, LT, and vitreous, are measured with the B-scanning mode using an equivalent refractive index of 1.3375. K values were measured using the Placido disc-based topography technique by projecting nine rings, each with 256 points, onto a 5.5 mm zone of the cornea, obtaining diameters of 2.0, 2.5, and 3 mm with a refractive index of 1.3375. For this study, a default 2.5 mm diameter was used [19,20].

### 2.5. Optimization of IOL Constants Using Linear Interpolation

**Figure 1:**
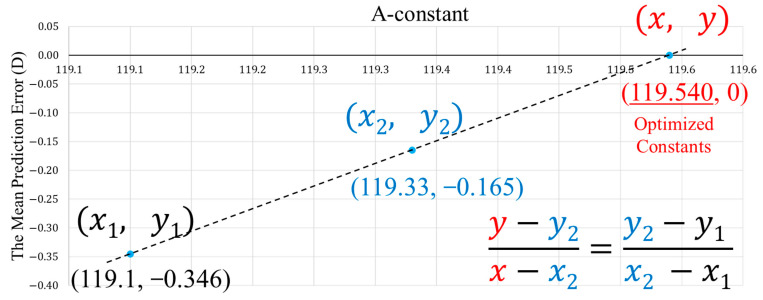
Conceptual diagram of linear interpolation applied for optimization of IOL constants. Two known A-constants and their corresponding mean prediction errors are plotted, and a straight line is interpolated. The optimized A-constant is determined as the *x*-coordinate at which MPE = 0.

To optimize the intraocular lens (IOL) constants, we applied LI based on two known constants and their associated MPEs. Specifically, the MPE was calculated at two A-constants (e.g., 119.1 and 119.33), and these were treated as coordinate points $\left(x_{1},y_{1}\right)$ and $\left(x_{2},y_{2}\right)$ on a Cartesian plane, where x represents the A-constant and y corresponds to MPE (as shown in Figure 1).

A straight line was interpolated between these two points, and the A-constant (i.e., MPE = 0) was derived using the LI formula:

$$y=y_{2}+\frac{\left(y_{2}-y_{1}\right)\left(x-x_{2}\right)}{x_{2}-x_{1}}$$

Solving this equation for x when y=0 yields the optimized A-constant. This calculation was implemented using Microsoft Excel’s “What-If Analysis” tool, specifically the Goal Seek function, which allowed for a convenient and accurate determination of the A-constant at which MPE = 0.

### 2.6. Correction of Predicted Refractions for Each Case Using Linear Interpolation

Once the optimized A-constant was determined, it was applied to individual eyes to correct their predicted refractions. Using the same LI principle, the predicted refraction at the optimized A-constant was calculated for each case using the following formula:

$$y^{\prime }=y_{2}^{\prime }+\frac{\left(y_{2}^{\prime }-y_{1}^{\prime }\right)\left(x-x_{2}\right)}{x_{2}-x_{1}}$$

Here, $y_{1}^{\prime }\;$ and $y_{2}^{\prime }$, respectively, denote the predicted refractions at the initial A-constants $x_{1}$ and $x_{2}$, and $y^{\prime }\;$ represents the corrected value. The optimized constant x obtained from the previous step was substituted into this formula to calculate the corrected predicted refraction for each eye.

In this study, we define these corrected predicted refraction values for each case as the “corrected values.” These values were used for subsequent accuracy analysis and statistical comparisons.

### 2.7. Inclusion and Exclusion Criteria

The inclusion criteria were eyes with an upper transconjunctival single-plane incision at 90°, an incision width of 2.5 to 2.7 mm, and a single side-port incision at 0° made using the Verion Image Guided System (Alcon); eyes with complete intracapsular fixation of the IOL; and eyes with no intraoperative or postoperative complications. Only one eye per patient was included, provided that it had a CVA of 20/40 or better, because ocular measurements are more alike between fellow eyes (causing a compounding of data) than between the eyes of different patients, and measurements from fellow eyes cannot be treated as if they were independent [12,13]. If both eyes met the inclusion criteria, the eye with the better CVA was selected. The exclusion criteria were a history of refractive surgery and corneal disease, such as pterygium or corneal endothelial disorders.

### 2.8. Primary Outcome Measures

The primary outcome measures were the optimized A-constants for each formula and device using the LI method. Additionally, predicted refractions were recalculated using the optimized A-constants with the ESCRS IOL Web Calculator to obtain “actual values” for each case.

Corrected values were then calculated using two methods. The first was a simple correction method, which involved subtracting the MPE from each predicted postoperative spherical equivalent (referred to as Method-S), where the MPE was calculated using the A-constant of 119.33. The second was a newly devised method using linear interpolation (Method-LI).

Furthermore, we calculated and compared the MAE, MedAE, the rates of cases with an absolute prediction error (APE) within 0.25 D and 0.5 D (abbreviated as “The rate <0.25D, 0.5 D”), and 95% confidence intervals (CIs) for both the actual and corrected values using the two methods.

### 2.9. Statistical Analysis

The sample size for comparisons using the Friedman test was estimated using G*Power 3.1.9.7, based on an initial subset of 30 cases enrolled early in the observation period. Assuming a significance level of 0.05 and a power of 0.8, the required sample size ranged from 18 to 24 eyes, depending on the analytical subgroup comparisons.

Statistical analyses were performed using Excel (version 2503, Build 16.0.18623.20208, 64-bit) from the Microsoft 365 suite, JAMOVI version 2.3.28.0 (the Jamovi Project) and R version 4.4.2. This retrospective study was conducted at a private clinic.

The normality of the APE was tested using the Shapiro–Wilk test for each combination of the device (ARGOS, OA-2000), calculation formula (EVO, Hill-RBF, Kane), and correction method (Method-S, Method-LI, and actual values). Repeated measures analysis of variance (ANOVA) was used to compare the MAE across groups showing normality, and the Friedman test, a non-parametric alternative, was used for non-normally distributed groups. Cochran’s Q test was used to compare rates <0.25 D and <0.5 D among the formulas and correction methods. When significant differences were found, the Durbin–Conover method or the McNemar test was used for post hoc analysis with Bonferroni correction.

### 2.10. Use of Generative AI

Generative AI (ChatGPT, OpenAI, May 2025) was used to assist with English editing, consistency checking, and phrase refinement during manuscript preparation.

## 3. Results

### 3.1. Participant Characteristics and Measurements

This study included 188 eyes from 188 Japanese patients (mean age ± standard deviation [SD]: 73.8 ± 7.1 years; male, 35.6%). The mean postoperative follow-up duration was 94.9 ± 8.3 days (range: 83–130 days). The measurements obtained using ARGOS and OA-2000 are summarized in Table 1. Significant differences were observed between the ARGOS and OA-2000 measurements for average AL, ACD, LT, CCT, HCD, and K values using the Wilcoxon signed-rank test or paired *t*-test (*p* < 0.001).

### 3.2. Optimized A-Constants and Mean Prediction Errors for Each Formula and Device

The default A-constants (119.1 and 119.33) and the optimized A-constants using the LI method and the corresponding MPEs are shown in Table 2. The optimized A-constants for each formula in ARGOS were 119.540 for EVO, 119.733 for Hill-RBF, and 119.563 for Kane. Similarly, for OA-2000, they were 119.388, 119.532, and 119.417, respectively.

All optimized A-constants resulted in actual MPEs close to 0, which proved the appropriateness of the LI method. There were clear differences between the A-constants recommended by the ESCRS Web Calculator for each formula, and it could be seen that the A-constants vary depending on the device, even with the same formula.

### 3.3. Comparative Analysis of Absolute Prediction Errors and Correction Methods for Each Formula and Device

#### 3.3.1. For All Cases

**Figure 2:**
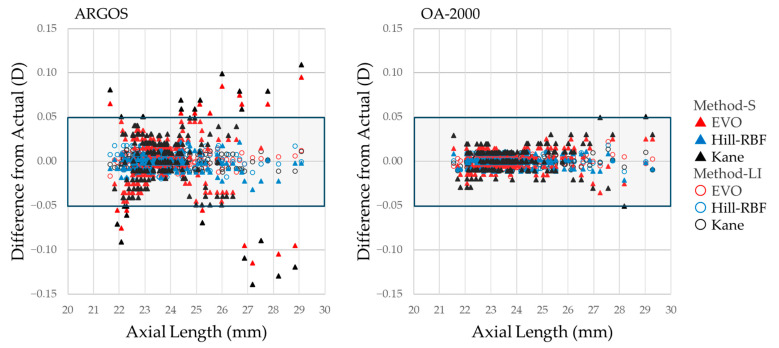
Scatter plots of the differences from Actual for Method-S (solid triangles) and Method-LI (open circles), plotted against axial length. The left panel shows data from ARGOS, and the right panel from OA-2000.

Table 3 summarizes the APEs across all calculation formulas, correction methods, and devices. The P-method indicates the *p*-value for comparisons between correction methods (Method-S, Method-LI, and actual values), and the P-formula indicates the *p*-value for comparisons among all calculation formulas. No significant differences were observed among the P-method values within each formula and device. However, in the formula comparison (P-formula) of ARGOS, the significance of the results varied depending on the correction method. Although no significant differences in MAE were observed among formulas with Method-S, significant differences emerged when using Method-LI or actual values. In the rate <0.5 D, although significant differences among formulas were observed under all correction methods, the results of post hoc analyses varied by method. Notably, the P-formula results were consistent between Method-LI and actual values across both devices. As shown in Figure 2, Method-LI consistently yielded prediction errors comparable to those of recalculated actual values across all formulas and both biometry devices, underscoring the reliability and reproducibility of the proposed optimization method. Post hoc power analysis (PHPA) achieved 100% power for all MAE comparisons.

Figure 2 shows scatter plots of the differences from Actual for Method-S and Meth-od-LI on the vertical axis, plotted against axial length on the horizontal axis. In particular, with ARGOS, many cases with short or long axial lengths exhibit large deviations from Actual with Method-S. In contrast, Method-LI shows minimal influence from axial length and yields values nearly identical to the Actual results.

#### 3.3.2. For Short-Axial-Length Cases

Table 4 presents the comparative analysis limited to eyes with short AL (AL < 22.5 mm). Previous studies have reported that ARGOS tends to measure slightly longer ALs in short eyes and shorter ALs in long eyes compared to other devices [13,14,15,16,17,18]. A similar tendency was observed in this study, resulting in a difference in the number of short AL cases between ARGOS and OA-2000. The prediction accuracy based on ARGOS measurements was generally lower than that of OA-2000, with the Hill-RBF formula particularly showing inferior performance compared to the other formulas. Under this short AL condition as well, the P-formula results were consistent between Method-LI and actual values across both devices. PHPA achieved >80% power for all MAE comparisons.

#### 3.3.3. For Long-Axial-Length Cases

Table 5 presents the comparative analysis limited to eyes with long axial length (AL > 25.5 mm). Under the long AL condition, the number of cases was similar between the two devices. With the exception of the EVO formula, the prediction accuracy based on ARGOS measurements was generally lower than that based on OA-2000 measurements, similarly to the trend observed in short AL cases. Moreover, when using ARGOS measurements, the Hill-RBF formula showed different results between Method-S and the other correction methods (*p* < 0.01), indicating that Method-S may be less reliable in long AL cases. As with other conditions, the P-formula results were consistent between Method-LI and actual values across both devices. PHPA achieved >80% power for all MAE comparisons.

## 4. Discussion

A distinctive feature of this study is that it not only proposed a method for IOL constants optimization using the LI method but also validated the accuracy of the method by recalculating predicted refractions with the optimized constants via the ESCRS IOL Web Calculator. Although the ESCRS Web Calculator provides predicted refraction values only to the second decimal place, the MPEs of actual values were nearly 0 across all formulas and devices, as shown in Table 2. This high accuracy may also be attributed in part to MPE’s ability to specify A-constants to the third decimal place. However, this finding strongly supports the accuracy and reliability of the LI method. This method requires predicted refractions based on two A-constants but enables A-constant optimization in Microsoft Excel. The optimized A-constants derived using this method can also be applied in clinical practice, making it practical for most medical facilities. Despite its simplicity, the method effectively optimized even advanced formulas such as EVO, Hill-RBF, and Kane. While formulas that utilize multiple proprietary constants, such as Haigis [21], were not evaluated in this study, the proposed method could potentially be adapted to optimize other constants, such as the Lens Factor used in the BU-II formula [3]. Although many modern IOL calculation formulas are now available via Web Calculators, these platforms do not permit user-defined optimizations of constants. As a result, the prediction accuracy is often unsatisfactory. This method could be a solution to this problem.

The usefulness of Method-LI was particularly evident in subgroup analyses based on AL, which have become increasingly emphasized in recent research [8,9,22,23,24]. In our study, the simple correction approach of Method-S often deviated in eyes with short or long ALs, with some results differing from the actual values. This variability could compromise the reliability of formula comparisons, particularly in subgroup analyses. Therefore, Method-S may be sufficient when the A-constant correction is minor; however, it becomes inadequate when the difference between the default and optimized A-constants is substantial, as in the case of CNA0T0. Although deriving actual values based on fully optimized A-constants is ideal, such an approach requires substantial time and effort, especially in a small center with limited personnel. In such settings, Method-LI may serve as a practical and sufficiently accurate alternative, yielding values that closely approximate the actual results.

**Figure 3:**
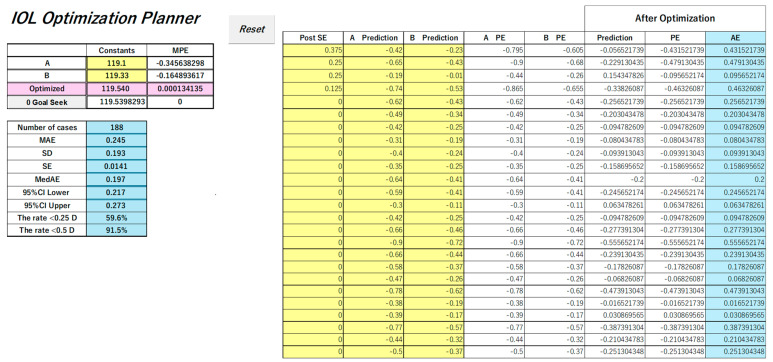
Screenshot of the Excel-based IOL Optimization Planner, which performs IOL constant optimization and case-by-case correction using linear interpolation. The interface includes input fields for predicted refractions at two constants, automatic calculation of optimized values, and corrected refractions for each case. Yellow cells indicate manual input fields for postoperative refractions and predicted values at two different IOL constants. Pink cells display the optimized IOL constant and its associated MPE. Blue cells present descriptive statistics and the corrected results calculated using Method-LI.

To further streamline the optimization process, we have also developed a semi-automated tool called the IOL Optimization Planner, implemented entirely in Microsoft Excel (Figure 3). This tool allows users to perform both constant optimization and case-specific correction with minimal manual effort, while also calculating descriptive statistics—such as the number of cases, MAE, MedAE, and confidence intervals—to facilitate comprehensive accuracy assessments without external software. The IOL Optimization Planner provides a practical, low-cost, and reproducible solution for clinicians, even in small-scale facilities without access to advanced statistical software. This tool also enables surgeons to promptly optimize constants and improve prediction accuracy when adopting new IOLs, devices, or calculation formulas. The same applies to the latest formulas published through Web Calculators.

Notably, the optimized A-constants differed across formulas and devices. For instance, the Hill-RBF formula required higher A-constants than EVO and Kane, and ARGOS consistently required higher values than OA-2000. As shown in Table 2, the optimized A-constants differed among formulas, consistent with the ESCRS Web Calculator recommendations for the CNA0T0. The reasons for these differences are unclear, as the details of the formula are not disclosed. The difference between devices may be partly attributed to ARGOS’s use of segmented refractive indices, which affects parameters such as ACD and LT [14,15,16,17,18,19]. However, Miyamoto et al. previously reported that even with formulas like SRK/T [25]—which do not incorporate these parameters—the optimized A-constants differed markedly between ARGOS and OA-2000 [19]. Although methods for correcting ALs obtained using equivalent refractive indices have been reported [26,27], the present study also revealed that small differences in ALs between the two devices corresponded to substantial differences in optimized A-constants. In our dataset, a device-related AL difference as low as 0.03 mm resulted in a difference exceeding 0.15 in the optimized A-constant. This discrepancy suggests that AL correction alone could not account for the variation and that other biometric parameters (e.g., K values) or device-specific factors likely play a substantial role. These findings underscore the need to optimize IOL constants individually for each formula and device.

While not the primary objective, this study also assessed the performance of the EVO, Hill-RBF, and Kane formulas. The predictive accuracy observed in our cohort was slightly better than the ranges reported in a recent systematic review [9,11]. Specifically, for the EVO formula, the MAE ranged from 0.245 to 0.253 D in our study compared to 0.27–0.35 D across previous studies, and the rate of <0.5 D ranged from 89.4% to 91.5%, exceeding the reported range of 75.2% to 86.4%. However, compared to our prior study [19] using the BU-II and Barrett True Axial Length (BTAL) formulas, the MAEs of this study were slightly higher (e.g., BU-II: 0.209–0.238 D and BTAL: 0.243 D), and the rates <0.5 D were marginally lower (e.g., BU-II: 92.5–95.0% vs. BTAL: 90.0%). These findings suggest that the EVO, Hill-RBF, and Kane formulas did not show a clear advantage over the BU-II and BTAL formulas in our population. Moreover, the Hill-RBF formula showed slightly lower accuracy than the others, particularly when used with ARGOS in short and long AL eyes. This may be due to the fact that none of the three formulas are currently adapted to ARGOS’s segmented refractive indices, unlike the BTAL formula. Future adaptations of these formulas could potentially improve their accuracy.

This study has several limitations. First, due to the scope of statistical processing that could be performed by a single investigator, only three IOL formulas and two devices were analyzed. However, the study design is feasible for implementation in multi-center clinical studies, making future expansion possible. Second, because the follow-up period was set at 3 months, a higher dropout rate among male patients was observed before this time point. In addition, male patients more frequently had Toric IOLs implanted in our facility due to a higher prevalence of astigmatism. These two factors jointly contributed to a gender imbalance in the analyzed sample. While this imbalance is unlikely to have significantly affected the optimization results, caution is warranted when interpreting the performance of formulas that incorporate sex-specific parameters—such as the Kane formula—in study populations with imbalanced gender distribution. Finally, this study was conducted at a single institution and involved a homogeneous, ethnically Japanese population. Therefore, we cannot rule out the potential influence of ethnicity. However, this study was deliberately designed to be easily replicable in diverse clinical settings regardless of institutional size. If validated in future studies across different regions and populations, the proposed method may contribute significantly to the global advancement of IOL power calculation accuracy.

## 5. Conclusions

Even when using the same IOL model, the optimization of constants (e.g., A-constants or Lens Factors) remains essential across different devices and formulas. The present method, which applies LI, allowed for simple yet precise optimizations and case-level corrections using only spreadsheet software. As new IOLs, devices, and formulas are introduced, the proposed method could remain applicable to a wide range of clinical scenarios. Ultimately, this approach may contribute to improving the accuracy of IOL power calculations in daily practice and assist physicians in conducting more accessible and reproducible optimization studies.

## 6. Patents

The IOL Optimization Planner introduced in this study has been officially filed for patent protection in Japan as of 16 June 2025 (Japanese Patent Application No. 2025-99851).

## Figures and Tables

**Table 1:** Summary of patient characteristics, postoperative outcomes, and ocular measurements using ARGOS and OA-2000.

Patient Characteristics			
Age	73.8 ± 7.1		
Sex (men, %)	35.6		
Follow-up duration (days)	94.9 ± 8.3		
**Measurements**			
	**ARGOS**	**OA-2000**	***p*** **-Value**
AL (mm)	23.81 ± 1.34	23.78 ± 1.37	<0.001
ACD (mm)	3.17 ± 0.34	3.08 ± 0.33	<0.001
LT (mm)	4.67 ± 0.40	4.70 ± 0.39	<0.001
CCT (μm)	528 ± 33	521 ± 33	<0.001 *
HCD (mm)	11.66 ± 0.38	11.56 ± 0.40	<0.001
K values (D)	44.47 ± 1.38	44.36 ± 1.38	<0.001 *

The Shapiro–Wilk test was used to assess normality. Depending on the results, either the Wilcoxon signed-rank test or paired *t*-test was applied to compare values between devices. * Tests marked with an asterisk were conducted using paired *t*-test. AL: Axial length; ACD: anterior chamber depth; LT: lens thickness; CCT: central corneal thickness; HCD: horizontal corneal diameter; K: keratometry.

**Table 2:** Summary of formula- and device-specific A-constant optimization and achieved mean prediction errors.

Device/Formula	Recommended A-Constant (CNA0T0)	Optimized A-Constant	A-Constant (119.1) MPE (D)	A-Constant (119.33) MPE (D)	Optimized A-Constant Actual MPE (D)
ARGOS					
EVO	119.1	119.540	−0.3456	−0.1649	−0.0006
Hill-RBF	119.26	119.733	−0.4431	−0.2820	−0.0012
Kane	119.33	119.563	−0.3763	−0.1893	−0.0006
OA-2000					
EVO	119.1	119.388	−0.2259	−0.0453	+0.0003
Hill-RBF	119.26	119.532	−0.3025	−0.1413	−0.0002
Kane	119.33	119.417	−0.2572	−0.0705	−0.0001

MPE: Mean prediction error; Actual: Predicted value using optimized A-constant.

**Table 3:** Comparative analysis of absolute prediction error and correction methods for each formula in ARGOS and OA-2000 (for all cases).

	Correction Method	EVO	Hill-RBF	Kane	P-Formula	Post Hoc
ARGOS (*n* = 188)						
MAE (D) ± SD	Method-S	0.243 ± 0.193	0.249 ± 0.205	0.252 ± 0.198	0.234	
	Method-LI	0.245 ± 0.193	0.248 ± 0.204	0.254 ± 0.194	<0.05 (1)	(1) H vs. K (<0.05)
	Actual	0.245 ± 0.193	0.248 ± 0.204	0.254 ± 0.193	<0.05 (2)	(2) H vs. K (<0.05)
	P-method	0.143	0.315	0.407		
The rate < 0.25 D (%)	Method-S	60.1	59	56.9	0.529	
	Method-LI	59.6	58.5	58	0.864	
	Actual	60.1	59.6	58.5	0.862	
	P-method	0.92	0.651	0.497		
The rate < 0.5 D (%)	Method-S	91	86.2	87.8	<0.05 (3)	(3) E vs. K (<0.05)
	Method-LI	91.5	86.2	88.3	<0.05 (4)	(4) E vs. H, E vs. K (<0.05)
	Actual	91.5	86.2	88.3	<0.05 (5)	(5) E vs. H, E vs. K (<0.05)
	P-method	0.368	1	0.716		
Standard Error	Method-S	0.0141	0.015	0.0144		
	Method-LI	0.0141	0.0149	0.0141		
	Actual	0.0141	0.0149	0.0141		
MedAE (D)	Method-S	0.205	0.208	0.209		
	Method-LI	0.197	0.21	0.205		
	Actual	0.198	0.2	0.205		
95% CI (MAE, %)	Method-S	0.216–0.271	0.220–0.279	0.224–0.281		
	Method-LI	0.217–0.273	0.219–0.278	0.226–0.281		
	Actual	0.217–0.273	0.219–0.277	0.226–0.281		
OA-2000 (*n* = 188)						
MAE (D) ± SD	Method-S	0.251 ± 0.192	0.244 ± 0.207	0.256 ± 0.198	0.147	
	Method-LI	0.252 ± 0.193	0.243 ± 0.206	0.258 ± 0.197	0.56	
	Actual	0.253 ± 0.194	0.244 ± 0.207	0.258 ± 0.198	0.553	
	P-method	0.114	0.616	0.894		
The rate < 0.25 D (%)	Method-S	60.1	59.6	57.4	0.692	
	Method-LI	58.5	59.6	57.4	0.804	
	Actual	59	61.2	59	0.744	
	P-method	0.247	0.223	0.223		
The rate < 0.5 D (%)	Method-S	89.4	88.8	88.8	0.931	
	Method-LI	89.4	88.3	88.8	0.819	
	Actual	89.4	88.8	89.4	0.931	
	P-method	NaN	0.368	0.607		
Standard Error	Method-S	0.014	0.0151	0.0145		
	Method-LI	0.0141	0.015	0.0144		
	Actual	0.0141	0.0151	0.0144		
MedAE (D)	Method-S	0.215	0.204	0.217		
	Method-LI	0.213	0.205	0.219		
	Actual	0.207	0.2	0.22		
95% CI (MAE, %)	Method-S	0.224–0.279	0.214–0.274	0.227–0.284		
	Method-LI	0.224–0.280	0.214–0.273	0.230–0.287		
	Actual	0.225–0.280	0.214–0.273	0.230–0.287		

The Friedman test and Cochran’s Q test were used for statistical analysis. When significant differences were detected, post hoc analyses were performed using the Durbin–Conover method for continuous variables and the McNemar test for categorical variables. Bonferroni correction was applied to adjust for multiple comparisons. NaN: Not applicable due to identical values in all groups. E: EVO formula; H: Hill-RBF formula; K: Kane formula; MAE: the mean absolute prediction error; rates <0.25 D and <0.5 D: the rates of cases with an absolute prediction error within 0.25 D and 0.5 D; MedAE: the median absolute prediction error; CIs: confidence intervals; Method-S: a method that corrects predicted values by subtracting the average prediction error for each case; Method-LI: a method that corrects predicted values using linear interpolation for each case; Actual: A value calculated based on optimized A constant obtained through linear interpolation.

**Table 4:** Comparative analysis of absolute prediction error and correction methods for each formula in ARGOS and OA-2000 (for short axial length of <22.5 mm).

	Correction Method	EVO	Hill-RBF	Kane	P-Formula	Post Hoc
ARGOS (*n* = 17)						
MAE (D) ± SD	Method-S	0.313 ± 0.186	0.373 ± 0.214	0.319 ± 0.194	0.251	(1, 2) NS. *
	Method-LI	0.293 ± 0.188	0.367 ± 0.213	0.296 ± 0.189	0.165	
	Actual	0.292 ± 0.190	0.369 ± 0.214	0.296 ± 0.190	0.147	
	P-method	<0.05 (1)	0.066	<0.05 (2)		
The rate < 0.25 D (%)	Method-S	41.2	35.3	35.3	0.819	
	Method-LI	47.1	35.3	41.2	0.449	
	Actual	47.1	35.3	35.3	0.549	
	P-method	0.368	NaN	0.368		
The rate < 0.5 D (%)	Method-S	82.4	64.7	76.5	0.368	
	Method-LI	88.2	64.7	82.4	0.197	
	Actual	88.2	64.7	82.4	0.197	
	P-method	0.368	NaN	0.368		
Standard Error	Method-S	0.045	0.052	0.047		
	Method-LI	0.0456	0.0517	0.0459		
	Actual	0.0462	0.0518	0.0462		
MedAE (D)	Method-S	0.305	0.432	0.301		
	Method-LI	0.287	0.43	0.306		
	Actual	0.28	0.44	0.3		
95% CI (MAE, %)	Method-S	0.217–0.408	0.263–0.483	0.219–0.418		
	Method-LI	0.196–0.389	0.257–0.476	0.199–0.394		
	Actual	0.194–0.380	0.259–0.479	0.199–0.394		
OA-2000 (*n* = 22)						
MAE (D) ± SD	Method-S	0.267 ± 0.154	0.260 ± 0.183	0.268 ± 0.151	0.967	
	Method-LI	0.265 ± 0.156	0.259 ± 0.182	0.265 ± 0.154	0.979	
	Actual	0.263 ± 0.156	0.260 ± 0.181	0.264 ± 0.154	0.992	
	P-method	0.197	0.511	0.446		
The rate < 0.25 D (%)	Method-S	50	54.5	54.5	0.913	
	Method-LI	54.5	59.1	54.5	0.895	
	Actual	54.5	63.6	54.5	0.607	
	P-method	0.368	0.223	1		
The rate < 0.5 D (%)	Method-S	95.5	77.3	90.9	<0.05 (1)	(1–3) NS. *
	Method-LI	95.5	77.3	95.5	<0.05 (2)	
	Actual	95.5	77.3	95.5	<0.05 (3)	
	P-method	NaN	NaN	0.368		
MedAE (D)	Method-S	0.245	0.245	0.237		
	Method-LI	0.244	0.241	0.233		
	Actual	0.24	0.24	0.24		
95% CI (MAE, %)	Method-S	0.199–0.335	0.179–0.341	0.201–0.335		
	Method-LI	0.195–0.334	0.179–0.340	0.197–0.333		
	Actual	0.194–0.332	0.180–0.340	0.195–0.332		

Repeated measures analysis of variance (ANOVA) and Cochran’s Q test were used for statistical analysis. Under these conditions, data were normally distributed, except for the Hill-RBF group of OA-2000, but Levene’s test confirmed homogeneity of variances. When significant differences were detected, post hoc analyses were conducted using a paired *t*-test and the McNemar test for categorical variables, with Bonferroni correction applied to account for multiple comparisons. NaN: Not applicable due to identical values in all groups. * Despite the significant result observed in the Cochran’s Q test, post hoc analysis using the McNemar test with Bonferroni correction did not reveal significant pairwise differences (*p* > 0.05). MAE: The mean absolute prediction error; rates <0.25 D and <0.5 D: the rates of cases with an absolute prediction error within 0.25 D and 0.5 D; MedAE: the median absolute prediction error; CIs: confidence intervals; Method-S: a method that corrects predicted values by subtracting the average prediction error for each case; Method-LI: a method that corrects predicted values using linear interpolation for each case; Actual: A value calculated based on optimized A constants obtained through linear interpolation.

**Table 5:** Comparative analysis of absolute prediction error and correction methods for each formula in ARGOS and OA-2000 (for long axial length of >25.5 mm).

	Correction Method	EVO	Hill-RBF	Kane	P-Formula	Post Hoc
ARGOS (*n* = 21)						
MAE (D) ± SD	Method-S	0.318 ± 0.278	0.378 ± 0.317	0.327 ± 0.278	0.538	(1) Method-S vs. -LI, Method-S vs. Actual (<0.01)
	Method-LI	0.311 ± 0.270	0.370 ± 0.316	0.315 ± 0.266	0.165	
	Actual	0.314 ± 0.268	0.371 ± 0.214	0.314 ± 0.264	0.147	
	P-method	0.18	<0.01 (1)	0.651		
The rate < 0.25 D (%)	Method-S	52.4	38.1	47.6	0.247	
	Method-LI	57.1	42.9	52.4	0.368	
	Actual	57.1	42.9	57.1	0.277	
	P-method	0.717	0.368	0.223		
The rate < 0.5 D (%)	Method-S	81	66.7	81	<0.05 (2)	(2) NS. *
	Method-LI	81	66.7	76.2	0.174	
	Actual	81	66.7	76.2	0.174	
	P-method	NaN	NaN	0.368		
Standard Error	Method-S	0.0607	0.0691	0.0606		
	Method-LI	0.0589	0.0689	0.058		
	Actual	0.0585	0.0685	0.0575		
MedAE (D)	Method-S	0.225	0.262	0.269		
	Method-LI	0.202	0.26	0.209		
	Actual	0.19	0.26	0.2		
95% CI (MAE, %)	Method-S	0.191–0.445	0.234–0.522	0.200–0.453		
	Method-LI	0.188–0.434	0.226–0.514	0.194–0.436		
	Actual	0.192–0.436	0.228–0.514	0.194–0.434		
OA-2000 (*n* = 21)						
MAE (D) ± SD	Method-S	0.325 ± 0.287	0.334 ± 0.330	0.293 ± 0.290	0.538	
	Method-LI	0.327 ± 0.288	0.334 ± 0.327	0.301 ± 0.283	0.953	
	Actual	0.330 ± 0.288	0.333 ± 0.325	0.301 ± 0.281	0.313	
	P-method	0.097	0.953	0.084		
The rate < 0.25 D (%)	Method-S	52.4	42.9	57.1	0.497	
	Method-LI	47.6	38.1	57.1	0.301	
	Actual	47.6	38.1	61.9	0.178	
	P-method	0.368	0.368	0.368		
The rate < 0.5 D (%)	Method-S	81	90.5	90.5	0.135	
	Method-LI	81	90.5	90.5	0.135	
	Actual	81	90.5	90.5	0.135	
	P-method	NaN	NaN	NaN		
Standard Error	Method-S	0.0627	0.072	0.0632		
	Method-LI	0.0628	0.0714	0.0617		
	Actual	0.0628	0.0709	0.0613		
MedAE (D)	Method-S	0.235	0.279	0.22		
	Method-LI	0.25	0.288	0.245		
	Actual	0.255	0.28	0.24		
95% CI (MAE, %)	Method-S	0.194–0.456	0.184–0.484	0.161–0.424		
	Method-LI	0.196–0.458	0.185–0.483	0.173–0.430		
	Actual	0.199–0.461	0.185–0.481	0.173–0.429		

The Friedman test and Cochran’s Q test were used for statistical analysis. When significant differences were detected, post hoc analyses were performed using the Durbin–Conover method for continuous variables and the McNemar test for categorical variables. Bonferroni correction was applied to adjust for multiple comparisons. NaN: Not applicable due to identical values in all groups. * Despite the significant result observed in the Cochran’s Q test, post hoc analysis using the McNemar test with Bonferroni correction did not reveal significant pairwise differences (*p* > 0.05). MAE: The mean absolute prediction error; rates <0.25 D and <0.5 D: the rates of cases with an absolute prediction error within 0.25 D and 0.5 D; MedAE: the median absolute prediction error; CI: confidence intervals; Method-S: a method that corrects predicted values by subtracting the average prediction error for each case; Method-LI: a method that corrects predicted values using linear interpolation for each case; Actual: a value calculated based on optimized A constant obtained through linear interpolation.

## Data Availability

The data supporting the findings of this study are available from the corresponding author upon reasonable request. Data are not publicly available due to privacy restrictions.
